# The Effect of Mechanical Loading on Mitophagy in Aged Myoblasts

**DOI:** 10.3390/cells15060522

**Published:** 2026-03-15

**Authors:** Evangelos Tolis, Eirini Chatzinikita, Athanasios Moustogiannis, Antonios Giannopoulos, Maria Maridaki, Michael Koutsilieris, Anastassios Philippou

**Affiliations:** 1 Department of Physiology, Medical School, National and Kapodistrian University of Athens, 115 27 Athens, Greece; evantolis@med.uoa.gr (E.T.); echatzin@med.uoa.gr (E.C.); amoustog@med.uoa.gr (A.M.); mkoutsil@med.uoa.gr (M.K.); 2 Section of Sports Medicine, Department of Community Medicine & Rehabilitation, Umea University, 901 87 Umea, Sweden; antonios.giannopoulos@umu.se; 3 Faculty of Physical Education and Sport Science, National and Kapodistrian University of Athens, 172 37 Athens, Greece; mmarida@phed.uoa.gr

**Keywords:** mechanical loading, mitophagy, senescence

## Abstract

**Background:** During aging, skeletal muscle mass constantly diminishes and myogenic potential declines. At the cellular level, a decline in mitochondrial function is a hallmark of the aging process and the deficiency of the mitochondrial network contributes to a progressive reduction in muscle mass. Autophagic clearance of mitochondria through the process of mitophagy is required to remove impaired or damaged mitochondria, while mitophagy is a key regulator of muscle maintenance. Dysfunctional degradation of mitochondria is increasingly associated with aging (mitophaging), while mechanical stimuli have been shown to ameliorate the aging-induced impaired muscle mass and function; however, less is known about the potential effects of mechanical loading on mitophaging. The aim of the present study was to investigate the effect of mechanical stretching on mitophagy in aged myoblasts, in vitro. **Methods:** Cell senescence was replicated using a multiple cell division model of C2C12 myoblasts. The control and aged cells were cultured on elastic membranes and underwent passive stretching using a mechanical loading protocol of 15% elongation for 12 h at a frequency of 1 Hz. Cell signaling and gene expression responses of mitophagy-associated and myogenic regulatory factors (MRFs) were assessed through immunoblotting and qRT-PCR of the cell lysates derived from stretched and non-stretched control and aged myoblasts. **Results:** Mitophagy factor AMP-activated protein kinase (AMPK), mitochondrial biogenesis stimulator peroxisome proliferator-activated receptor gamma coactivator 1-alpha (PGC-1a), and mitophagy/mitochondrial biogenesis factor Parkin were downregulated in control stretched myoblasts compared to non-stretched cells, while the specific mechanical loading protocol used also reduced the phosphorylation of unc-51-like autophagy-activating kinase 1 (p-ULK1) (*p* < 0.05), as well as the expression of myogenic factor 5 (Myf5) and myogenic factor 4 (myogenin) (*p* < 0.001). Interestingly, this mechanical loading resulted in increased PGC-1a and Parkin expression (*p* < 0.05) and induced the previously undetected BCL2 interacting protein 3-like (BNIP3L/NIX) and AMPK expression and p-ULK1 activation in the aged myoblasts. In addition, mechanical stretching differentially affected the expression of MRFs in aged cells, upregulating the early differentiation factor, Myf5 (*p* < 0.01), while downregulating the late differentiation factor myogenin (*p* < 0.001). **Conclusions:** These findings suggest the beneficial effects of mechanical loading on the impaired mitophagy and early differentiation in aged myoblasts, as indicated by the mitophagy initiation and the promotion of mitochondrial biogenesis in these cells. The mechanical loading-induced downregulation of mitophagy and myogenesis in the control myoblasts might indicate their loading-specific differential responses compared to the aged cells.

## 1. Introduction

Cell survival and adaptation in response to cellular stress need high-energy production, which is achieved via the function of organelles called mitochondria [1,2]. These cellular organelles have the capacity to change their function and morphology in response to different stimuli, originating both from within, as well as outside the cell [3]. Mitochondria are synthesized anew, arising from the growth and division of pre-existing mitochondria through the process known as mitochondrial biogenesis. This process requires an increase in cellular respiration, metabolism, and ATP production [4,5], while mitochondrial selective clearance is realized through autophagic degradation, known as mitophagy [6]. The interaction and regulation between the opposing processes of mitochondrial biogenesis and mitophagy are essential for the cellular adaptation to environmental and intracellular signals, e.g., mechanical loading and metabolic stress in skeletal muscle cells [1,7].

Mitochondrial biogenesis and mitophagy are balanced through the opposing processes of fusion (elongation) and fission (division) [8]. Specifically, fusion promotes mitochondrial DNA (mtDNA) complementation and thus the repair of defective mitochondria, while fission removes those parts of mitochondria that have been damaged, preventing major wasting of the mitochondrial network in its entirety [8,9]. The specific part of the mitochondrion that has been compromised is separated from the rest and is marked via ubiquitylation [10]. This works as a signal for mitophagy to commence through activation of mitochondrial autophagic receptors, which in turn forms autophagic membranes [11]. These membranes encase the damaged parts of the mitochondrion (or an entire mitochondrion), creating what is known as an autophagosome, which will fuse with a lysosome, finally leading to mitochondrial degradation [11,12].

**Figure 1:**
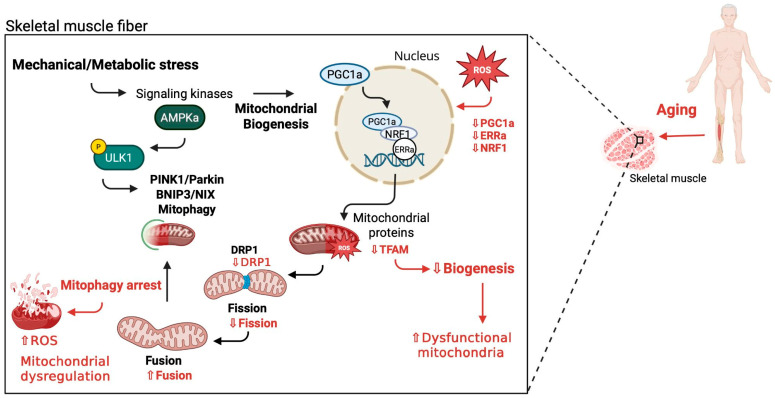
The effect of mechanical loading on mitophaging. Aged skeletal muscle presents a higher reactive oxygen species (ROS) production, which leads to a decreased expression and subsequent inability of mitochondrial biogenesis regulator PGC-1α to enter the cell nucleus. ERRa and NRF1 expression is also diminished due to increased ROS. ROS further accumulate in the mitochondria and, along with the reduction in the TFAM expression, result in a decrease in mitochondrial biogenesis and build-up of dysfunctional mitochondria. Moreover, DRP1 expression also declines, thus decreasing mitochondrial fission and increasing mitochondrial fusion, eventually inhibiting mitophagy, while further increases in ROS result in mitochondrial dysregulation. These aging-associated pathways/events are indicated in red. On the other hand, mechanical stimuli and/or metabolic stress in aged skeletal muscle can activate AMPK, which phosphorylates ULK-1, which in turn induces the activation of the mitophagy receptor, BNIP3L/NIX, hence enhancing mitophagy. AMPK activation through mechanical stress also contributes to the nuclear translocation of PGC-1α, which in turn augments mitochondrial biogenesis. These mechanical loading-induced pathways/events that favor mitochondrial function and biogenesis are depicted in black. PGC-1α: peroxisome proliferator-activated receptor gamma coactivator 1-alpha; ERRα: estrogen-related receptor α; NRF1: nucleus respiratory factor 1; TFAM: transcription factor 1, mitochondrial; DRP1: dynamin-related protein 1; AMPK: adenosine monophosphate-activated protein kinase; ULK-1: unc-51-like autophagy-activating kinase 1; BNIP3L/NIX: BCL2 interacting protein 3-like.

In skeletal muscle, the transition from each of these steps to the next, as well as the molecular mechanisms that induce the individual processes, have not yet been decrypted [7]. Moreover, while several mechanisms regulate quality control in mitochondria, aging seems to adversely affect the interplay between mitochondrial biogenesis and mitophagy the most [13] (Figure 1). The accrual of protein damage during aging seems to functionally impair the mitochondria in skeletal muscle. When multiple damaged or dysfunctional mitochondria are accumulated in the aging muscle, mitophagy may become impaired [14,15]. Various studies on aged skeletal muscle have revealed higher levels of fission (Fis-1, Opa-1) compared to fusion proteins (Mfn1, Mfn2, and Drp-1) [16]. To compensate for this age-associated increase in the fission process, it has been suggested that mitochondria elongate due to intrinsic defects for promoting fusion [3]. Additionally, the mitochondrial dysfunction observed in aged skeletal muscle is not entirely caused by impaired mitophagy but also as a result of reduced mitochondrial biogenesis, as these two cellular processes are inextricably linked [1,17].

The impact that aging has on skeletal muscle mitophagy pathways has only scarcely been examined [18]. It could be assumed that the aging-induced mitochondrial dysfunction and fragmentation would result in increased mitophagy signaling to promote the removal of these dysfunctional organelles, and previous studies have reported increased expression of mitophagy proteins, such as Pink1 and Parkin, in aged muscle. The status of these proteins could indicate whether the mitophagy cascades are activated; nevertheless, their expression has not yet been examined in senescent muscle cells [19,20,21]. In addition, although the Pink1–Parkin pathway remains the most studied ubiquitylation mechanism in mitochondria, its necessity for skeletal muscle mitophagy has not been defined [6]. Specifically, Parkin is a basic component of signaling axis both in mitophagy and mitochondrial biogenesis, but limited evidence supports the existence of Pink1–Parkin-mediated mitophagy in skeletal muscle [10]. Nevertheless, a decreased Parkin expression and ubiquitylation in aged mouse muscles has been reported, suggesting that the Pink1–Parkin mitophagy response could occur in senescence [10].

Mitochondrial fission and mitophagy coordination are suggested to be regulated by AMPK [22]. Early studies have also shown that ULK-1 phosphorylation (Ser 555), mediated by AMPK, facilitates mitophagy [23]. When AMPK is inactive, basal mitophagy may occur, indicating that other, currently unknown, pathways could regulate mitophagy as a response to various stimuli [7]. In addition, AMPK activates both Parkin and Pink1 for mitophagy induction but not in C2C12 skeletal muscle myotubes. Despite the fact that the Pink1–Parkin pathway induces mitophagy, it is not necessary for the activation of AMPK-mediated mitophagy in skeletal muscle [10,21,24]. Lately, AMPK has emerged as an important mitophagy regulator in skeletal muscle, as its activation enhances mitochondrial fission before promoting the engulfment of damaged/dysfunctional mitochondria by the autophagosomes [22,25]. In mitochondrial biogenesis, the activation of AMPK appears to act as a promoter via the phosphorylation of PGC-1α [4].

During aging, PGC-1α has a major role in crosstalk modulation of signaling pathways that control mitochondrial quality, and it also regulates mitochondrial biogenesis [13]. For instance, the expression of Mfn2, a major player in the PINK–Mfn2–Parkin axis, is controlled by PGC-1α [26]. This is important because the mitophagic process is significantly impacted by alterations in mitochondrial dynamics. A disruption in mitochondrial homeostasis due to a PGC-1α-reduced expression could lead to increased ROS production, FOXO nuclear sequestration, and mitophagy during aging [13,27].

In addition, BNIP3L/NIX, an outer membrane protein, functions as a receptor for mitophagy and has been found to play a crucial role in receptor-mediated mitophagy, facilitating muscle health [28,29,30]. Indeed, deficiencies in this protein in muscle cells have been associated with increased inflammation and an impairment in mitochondrial function during aging [30,31,32]. Moreover, in the absence of Pink1/ Parkin, BNIP3L/NIX appears to be a key factor in mitochondrial clearance during muscle cell differentiation [33].

Mitophagy also plays a major role in repairing/forming new muscle cells through myogenesis and muscle regeneration [1]. Myogenesis is induced through different signaling pathways regulating a variety of factors, particularly the myogenic regulatory factors (MRF’s), Myf5 and myogenin, which together control the growth and development of skeletal muscle [18,34]. Interestingly, mitophagy and myogenesis are important muscle cellular processes, quite possibly associated, and they have both been shown to become impaired during the aging process [1].

Utilizing in vitro models to simulate senescence in skeletal muscle cells could greatly improve our knowledge regarding the cellular and molecular aspects of aging-induced alterations [35]. Moreover, mechanical loading is able to cause adaptations in skeletal muscle cells, highlighting muscle’s plasticity and its ability to alter its mass and contractile phenotype [14,36] (Figure 1). While mechanical stimuli have been shown to improve the impaired myogenesis in aged muscle [37], their effect on the myogenesis-associated mitophagy and the dysfunctional degradation of mitochondria associated with aging (mitophaging) is unclear [38]. Hence, in vitro mechanical loading models in aged muscle cells can be of particular importance for investigating the role of mechanical stimuli in mitophaging [36,39,40]. Therefore, the aim of this study was to examine the effects of mechanical stretching on mitophagy and myogenic potential in aged myoblasts, in vitro.

## 2. Materials and Methods

### 2.1. Cell Cultures

C2C12 mouse cell line of skeletal myoblasts was acquired from the American Type Culture Collection (Manassas, VA, USA) and cultured as described in detail elsewhere [41]. Collagen 1-coated, flexible-bottomed six-well plates (Flex 1 Culture Plates Collagen 1; Flexcell International. Hillsborough, NC, USA) were used to culture the myoblasts in order to mechanically load them as described elsewhere [37,42]. Briefly, all experiments were conducted in proliferating myoblasts maintained in growth medium (10%FBS, 1% pen/strep). A differentiation medium was not used at any stage of the experimental procedures, and multinucleated myotubes were at no point generated in the present study.

### 2.2. Cellular Senescence

For the purpose of examining the effect of mechanical loading on the aged C2C12 myoblasts, an already characterized muscle cell aging model was used [43]. Briefly, at least 80 myoblast multiple doublings were completed for achieving senescence in the C2C12 cells. Non-senescent control myoblasts were used at passage 14 (P14), whereas aged (senescent) myoblasts were used at passage 38.

### 2.3. Mechanical Loading

Control and aged C2C12 myoblasts were subjected to passive cyclic stretching utilizing the FX-5000 strain unit (Flexcell International, Hillborough, NC, USA), as previously described [44]. The cells underwent a stretching protocol of 15% elongation at a frequency of 1 Hz for 12 h. For both the aged and the control myoblasts, an additional non-stretching condition was used.

### 2.4. Cell Lysis, RNA Extraction, Reverse Transcription, and Real-Time RT-PCR

NucleZOL (Mecherey-Nagel, Duren, Germany) was used for C2C12 myoblast lysis and the collection of cell extracts performed as previously described [42,44]. mRNA expression of the genes of interest was examined in mechanically loaded and unloaded myoblasts after total RNA isolation, reverse transcription, and semi-quantitative real-time PCR (RT-PCR) as stated elsewhere [42,44]. The sequences of the primer sets used for the examined factors are as follows: GAPDH_fwd CAA CTC CCT CAA GAT TGT CAG CAA; GAPDH_rev GGC ATG GAC TGT GGT CAT GA; Myf5_fwd CTA TTA CAG CCT GCC GGG AC; Myf5_rev CTC GGA TGG CTC TGT AGA CG; myogenin_fwd AGG AGA GAA AGA TGG AGT CCA GAG; and myogenin_rev TAA CAA AAG AAG TCA CCC CAA GAG.

### 2.5. Protein Extraction and Immunoblotting Analysis

Total protein extracts were collected from C2C12 myoblasts using RIPA Buffer (Sigma-Aldrich, St.Louis, MO, USA, R0278-50ML) mixed with phosphatase inhibitor (Cell Signaling Technology, Danvers, MA, USA, #5870S) and protease inhibitor (Cell Signaling Technology, #5871S) Cocktail. The extracts underwent electrophoresis (SDS-PAGE), were transferred to a polyvinylidene fluoride (PVDF) membrane, and were blotted with primary [AMPK Rabbit Ab (1:100; Cell Signaling Technology, #5831); p-Ulk1 (Ser 555) Rabbit Ab (1:1000; Cell Signaling Technology, #5869), BNIP3L/Nix Rabbit Ab (1:1000; Cell Signaling Technology, #12396), Parkin Mouse Ab (1:1000; Cell Signaling Technology, #4211); PGC1 alpha Rabbit Ab (1:1000; Novus Biologicals, NBP1-04676); β-Actin Mouse Ab (1:1000; Santa Cruz Biotechnology, Santa Cruz, CA, USA, sc-47778)]; and secondary antibodies [anti-rabbit IgG (goat anti-rabbit, 1:2000; Santa Cruz Biotechnology, Santa Cruz, CA, USA sc-2004) or anti-mouse IgG (goat anti-mouse, Santa Cruz Biotechnology, Santa Cruz, CA, USA 1:2000; sc-2005)] for the immunodetection of the protein of interest. The Invitrogen iBright 1500 Imaging System (Thermo Fisher Scientific, Mississauga, ON, Canada) and the iBright Analysis Software (v. 5.5.0) were used for the semi-quantification of the band intensity. The protein extraction and immunoblotting analysis procedures followed had been described in detail previously [41,42,44,45]. Only bands corresponding to the expected molecular weight of each target protein were included in the immunoblotting analysis. Western blots were performed in two independent biological experiments per condition.

### 2.6. Statistics

The data are presented as mean ± standard error (SE). The SPSS^TM^ 30 statistical analysis software was utilized to perform Student’s *t*-test and/or the Mann–Whitney test. Statistical significance was determined at *p* < 0.05.

## 3. Results

### 3.1. Effect of Mechanical Loading on Mitophagy Signaling in C2C12 Myoblasts

**Figure 2:**
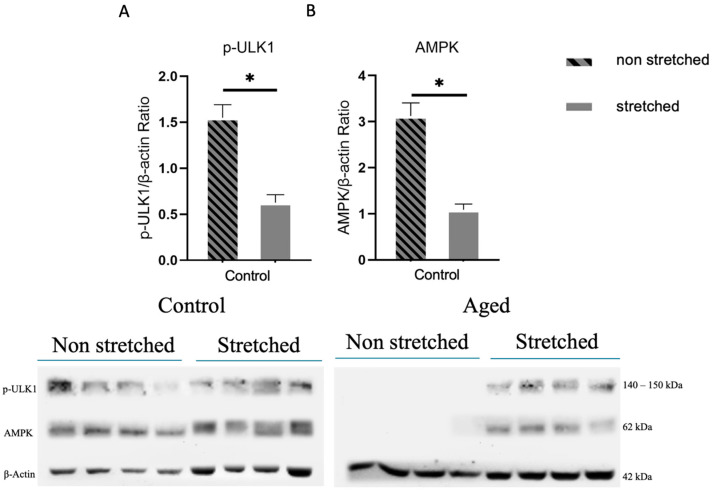
The effects of mechanical loading on the mitophagy signaling proteins. Representative Western blots and immunoblotting quantification of p-ULK1 (**A**), AMPK (**B**) in the control and aged myoblasts compared to non-stretched cells. The induction of previously undetected p-ULK1 and AMPK was found after stretching in the aged myoblasts. β-actin was used as the internal standard to normalize target proteins expression/activation levels (significantly different: * *p* < 0.05). Quantification was performed exclusively on the bands corresponding to the expected molecular weights. p-ULK1: phosphorylated unc-51-like autophagy-activating kinase 1; AMPK: adenosine monophosphate-activated protein kinase.

The effects of mechanical loading on mitophagy signaling were examined by the phosphorylation of p-ULK1 and the expression levels of AMPK protein in the C2C12 cells. It was found that in the control myoblasts, the stretching protocol resulted in significant decreases in both p-ULK1 (Figure 2A) and AMPK expression (Figure 2B) compared to their non-stretched counterparts. Interestingly, in the aged cells, the mechanical loading protocol induced the activation/expression of these two proteins, which were undetected in the non-stretched cells.

### 3.2. Effect of Mechanical Loading on Mitophagy Induction in C2C12 Myoblasts

**Figure 3:**
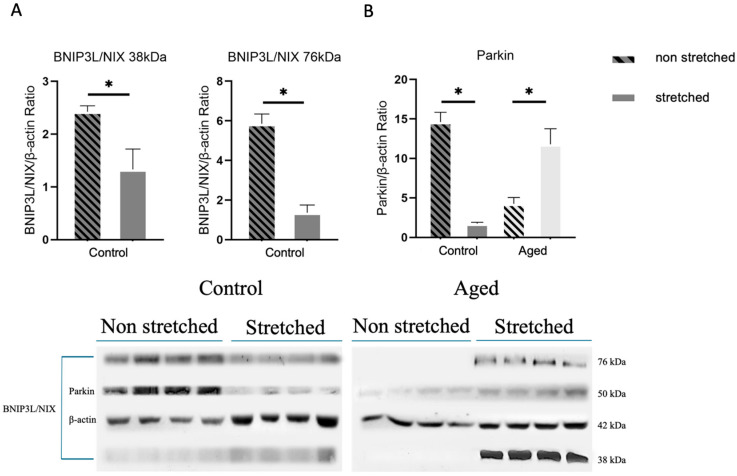
The effects of mechanical loading on the expression of mitophagy-associated proteins. Representative Western blots and immunoblotting quantification of BNIP3L/NIX (**A**) and Parkin (**B**) in the control and aged myoblasts compared to the non-stretched cells. The induction of previously undetected BNIP3L/NIX in the aged myoblasts was observed after mechanical stretching. The 38 kDa and 76 kDa bands correspond to the monomeric and dimerized forms of BNIP3L/NIX, respectively and were quantified separately. β-actin was used as the internal standard to normalize target protein expression (significantly different: * *p* < 0.05). Quantification was performed exclusively on the bands corresponding to the expected molecular weights. BNIP3L/NIX: BLC2/adenovirus E1B 19 kDa interacting protein 3-like; Parkin: parkin RBR E3 ubiquitin protein ligase.

The induction of mitophagy was further assessed via the expression levels of the BNIP3L/NIX receptor and Parkin. The stretching protocol caused significant downregulation of BNIP3L/NIX and Parkin in control cells compared to non-stretched myoblasts (Figure 3A), while in the aged myoblasts, mechanical loading induced the expression of the previously undetected BNIP3L/NIX mitophagy receptor and the upregulation of Parkin (Figure 3B). Notably, for BNIP3L/NIX, the 38 kDa band corresponds to its monomeric form, while the 76 kDa band represents its dimerized form, associated with functional activation and mitochondrial membrane insertion. Additionally, Parkin expression in aged non-stretched cells was near the detection threshold and relative fold changes post-loading are amplified due to low basal levels.

### 3.3. Effect of Mechanical Loading on Mitochondrial Biogenesis in C2C12 Myoblasts

**Figure 4:**
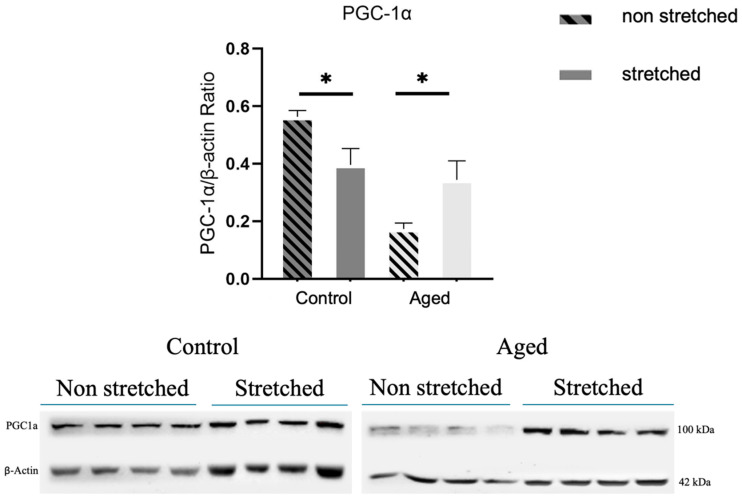
The effects of mechanical loading on the expression of the key regulator of mitochondrial biogenesis, PGC-1α. Representative Western blots and immunoblotting quantification of PGC-1α expression compared to non-stretched cells in the control and aged myoblasts. β-actin was used as the internal standard to normalize target protein expression (significantly different: * *p* < 0.05). Quantification was performed exclusively on the bands corresponding to the expected molecular weights. PGC-1α: Peroxisome proliferator-activated receptor gamma coactivator 1-alpha.

To investigate the effect of mechanical loading on mitochondrial biogenesis, the expression of PGC-1α, a key factor in this process, was examined. Control cells exhibited a significant decline in the PGC-1α expression after stretching compared to non-stretched cells (Figure 4). On the contrary, the aged cells showed a significant upregulation expression of this factor compared to their non-stretched equivalents (Figure 4).

### 3.4. Effect of Mechanical Loading on Myogenic Regulatory Factors in C2C12 Myoblasts

**Figure 5:**
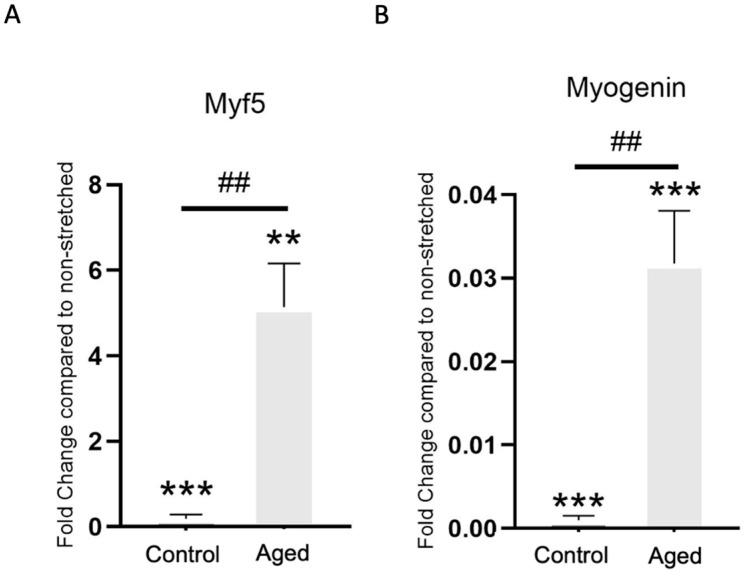
Mechanical loading-driven transcriptional changes in myogenic regulatory factors, Myf5 (**A**) and myogenin (**B**), in control and aged C2C12 myoblasts. The transcriptional response of each gene examined was normalized to the corresponding GAPDH and is expressed as fold change compared to the non-stretched condition (mean ± SE of three independent experiments performed in triplicate. ## *p* < 0.01, significant differences between conditions; ** *p* < 0.01, *** *p* < 0.001, significantly different compared to non-stretched cells). Myf5: myogenic factor 5; myogenin: myogenic factor 4.

The myogenic potential of the control and aged myoblasts was investigated via the mRNA expression of the early myogenic factor, Myf5, as well as the late myogenic factor, myogenin. Myf5 demonstrated a decreased mRNA expression after mechanical loading in control cells compared to non-stretched cells, while the opposite effect of mechanical stretching was observed in the aged myoblasts (Figure 5A). Contrastingly, the late myogenic differentiation factor, myogenin, presented a decline in mRNA expression in both the control and aged cells compared to the non-stretched cells (Figure 5B). All analyses were performed in proliferating myoblasts maintained in growth medium, without induction of differentiation.

## 4. Discussion

This study investigated the effect of mechanical loading on the process of mitophagy and myogenic regulation in senescent myoblasts, in vitro. Our group has previously characterized how aging affects myogenic lineage [42], as well as examined the mechanical loading protocol(s) that most effectively induces myogenic differentiation and survival in these cells [37]. However, less was known concerning the influence of mechanical loading on myogenesis- and aging-associated mitophagy (mitophaging) [38]. Herein, we found that implementing a specific mechanical stretching protocol, which has previously been characterized as high-strain loading [37], did induce significant alterations in multiple mitophagy-related factors, modulating mitophagy signaling pathways, mitochondrial biogenesis-associated regulators, and early differentiation markers in aged myoblasts, while it downregulated these cellular processes in the control cells.

In the signaling of mitophagy, AMPK possesses a distinct regulatory function to maintain control of mitochondrial quality in muscle cells [46]. On the other hand, dependent on AMPK activity and essential for targeting the damaged mitochondria to lysosomes, p-ULK1 is a critical factor in mitophagy process in skeletal muscle [47]. The present study revealed the downregulation of p-ULK1 and AMPK in the control (non-senescent) cells and the induction of previously undetected expression/activation of these proteins in the aged cells, as a result of the loading protocol used. These findings suggest that mechanical loading modulates key regulators involved in mitophagy initiation signaling, indicating the possible effects this loading exerts on mitophagy signaling. Interestingly, these effects appear to be beneficial for the mitophagy pathway in the aged cells and detrimental in the non-senescent cells.

Similarly, a significant reduction in the expression of the mitophagy receptor BNIP3L/NIX, which mediates the clearance of damaged mitochondria [28], and of Parkin, which likewise facilitates the degradation of damaged mitochondria [19], were observed in the control cells, along with an increased expression of Parkin and the detection of the previously undetected BNIP3L/NIX receptor in the aged cells. The increase in Parkin expression in the aged cells is noteworthy, since this factor also plays an important role in basal mitophagy [33,48]. Overall, the same pattern of response to mechanical loading, as in mitophagy signaling, was further observed with the aged cells exhibiting upregulation of these crucial for mitophagy activation factors and the control cells showing a decline in the expression levels of the same proteins, confirming that the specific loading protocol used differentially affected mitophagy-associated pathways in the aged cells compared to the control cells.

Moreover, PGC-1α, a key regulator of mitochondrial biogenesis [13], was shown in this study to also respond to mechanical loading, with increased expression in the aged cells and its downregulation in the control cells, providing further evidence of a loading-specific differential response of the aged cells compared to the controls. These findings suggest that high-strain loading can induce mitochondrial biogenesis in senescent muscle cells while diminishing it in control cells. Overall, it can be postulated that the cell stretching protocol utilized was associated with the activation of signaling molecules implicated in mitophagy initiation, induction, and mitochondrial biogenesis, thus facilitating a possibly beneficial mitophagy response and subsequently mitochondrial regeneration by augmenting mitochondrial biogenesis in the aged cells but generating a detrimental effect in mitochondrial renewal by negatively impacting mitophagy and mitochondrial biogenesis pathways in the control cells.

In addition, the present study examined the effects of mechanical loading on myogenic differentiation by checking the mRNA expression of two key muscle cell differentiation factors responsible for controlling muscle growth and development, the MRFs Myf5 and myogenin [18,34]. It was found that, while the early differentiation factor, Myf5, showed an augmented expression in response to mechanical stretching in the senescent cells compared to their non-stretched counterparts, the opposite response was observed in the control cells, revealing that the stretching protocol employed may also positively influence muscle cell differentiation in senescence whilst downregulating this process in control cells. The late differentiation factor, myogenin, presented a reduced expression in both the control and the aged myoblasts after mechanical loading, implying that the high-strain protocol implemented could disturb the completion of myogenic differentiation not only in the context of senescence, but also in non-senescent cells. It should be emphasized that these observations were obtained in proliferating myoblasts under growth medium conditions and therefore reflect modulation of myogenic regulatory pathway rather than differentiation outcomes.

Overall, the aforementioned findings indicate that mechanical loading can indeed affect mitophagy in both control and aged myoblasts by influencing the expression of key factors that regulate the mitophagy activation and signaling. Furthermore, mechanical loading appeared to also have an effect on mitochondrial renewal, as it impacts the expression of a major regulator of mitochondrial biogenesis. It has been previously reported that aging diminishes muscle cell sensitivity to mechanical stimuli, reducing the efficiency of mitophagy [49,50]. On the other hand, non-senescent muscle cells are more sensitive to mechanical stimuli, and high amounts of loading can negatively affect mitophagy [47]. Intriguingly, in this study, the manifestations of mechanical loading in the regulation of all the factors examined indicate that a high-strain loading protocol is potent enough to enhance mitophagy in aged cells, but it downregulates it in control cells. It therefore could be assumed that the protocol used unfavorably altered mitophagy-related pathway responses in the control cells while enhancing them in aged cells, suggesting a differential response of myoblasts to mechanical loading dependent on whether cellular senescence has occurred. Similarly, the high-strain mechanical loading exerted different effects on myogenic potential of senescent vs. control cells, inducing beneficial effects on early-stage differentiation in aged myoblasts and detrimental effects in control myoblasts, while disrupting late-stage myogenic differentiation regardless of cell senescence.

A limitation of the present study is that mitophagy and mitochondrial biogenesis were assessed at the level of signaling and protein expression rather than through direct measurement of mitophagic flux or mitochondrial turnover. Therefore, the present findings reflect a modulation of mitophagy-related regulatory pathways rather than direct quantification of functional mitophagy activity. Future studies employing flux-based assays and morphological approaches will be required to further validate the functional consequences of the observed molecular changes. Another limitation is the use of deformable collagen-coated, flexible-bottomed culture plates, required for cyclic mechanical loading. Due to the nature of the deformable membranes of those plates, high-resolution fluorescence imaging or ultrastructural transmission electron microscopy analysis could not be performed within the same experimental setup.

## 5. Conclusions

Mitochondria produce the necessary energy for cell survival and adaptation and have been shown to respond to intracellular or extracellular stimuli. The cell’s fate is determined by the interaction between mitochondrial biogenesis and selective clearance known as mitophagy. Aging is known to adversely affect both mitophagy (mitophaging) and biogenesis in muscle cells; however, mechanical stimuli have been shown to cause adaptations in skeletal muscle and improve myogenesis, though their effects on myogenesis-associated mitophagy remain inconclusive. This study utilized an in vitro mechanical loading protocol to assess the mitophagy responses in control and senescent myoblasts and revealed a differential modulation of mitophagy signaling and initiation, as well as mitochondrial biogenesis proteins dependent on cellular senescence. The findings of the study indicate that mechanical loading influences key molecular regulators associated with impaired mitophagy and early differentiation in aged myoblasts, as suggested by mitophagy initiation and the promotion of mitochondrial biogenesis in these cells, in contrast to the downregulation of mitophagy and myogenesis in the control myoblasts, indicative of loading-specific differential responses of these cells compared to the aged cells. These findings might serve as a valuable source for further research into skeletal muscle mitophaging and the development of different experimental models for studying the effects of mechanical loading in senescent skeletal muscle mitochondrial function.

## Data Availability

The original contributions presented in this study are included in the article. Further inquiries can be directed to the corresponding author.
